# Thermo-Chemical Modification of Cellulose for the Adsorptive Removal of Titan Yellow from Wastewater

**DOI:** 10.3390/molecules28093955

**Published:** 2023-05-08

**Authors:** Ubaid Ur Rahman, Muhammad Humayun, Abbas Khan, Saima Farooq, Muhammad Sadiq, Mohamed Bououdina, Nasrullah Shah

**Affiliations:** 1 Department of Chemistry, Abdul Wali Khan University Mardan, Mardan 23200, Pakistan; ubaidur733@gmail.com (U.U.R.); nasrullah@awkum.edu.pk (N.S.); 2 Department of Mathematics and Sciences, Faculty of Humanities and Sciences, Energy, Water, and Environment Lab, Prince Sultan University, Riyadh 12435, Saudi Arabia; mbououdina@psu.edu.sa; 3 Department of Biological Sciences and Chemistry, College of Arts and Sciences, University of Nizwa, Nizwa 616, Oman; saima@unizwa.edu.om; 4 Department of Chemistry, University of Malakand, Chakdara 18800, Pakistan; sadiq@uom.edu.pk

**Keywords:** dyes removal, adsorption, thermo-chemical modification, thermodynamics, kinetics

## Abstract

This research work focuses on the isolation and thermo-chemical modification of cellulose and its application as an adsorbent for the removal of organic pollutants. The used cellulose was collected from a locally available plant (*Olive Europa*) commonly called Zaitoon. The stem branches of Zaitoon were collected and then kept in water for 40–45 days at room temperature to extract the cellulose fibers. These cellulose fibers were then kept in the Soxhlet apparatus for washing in n-hexane for 72 h. The purified cellulose was divided into three parts: one part was subjected to thermal activation (TAC), the second was modified chemically (CMC) with Benzyl Chloride, while the last one remained un-functionalized (UFC). All the three forms of cellulose were characterized via FTIR and SEM, then utilized for the removal of Titan Yellow (TY) dye from aqueous media via adsorption process by varying the contact time, temperature, concentration of dye and type, and dose of adsorbent. The adsorption efficiencies of all adsorbents were compared under different experimental variables. Thermally activated cellulose showed the best results for the removal of TY compared with other materials. The calculated removal percentage of TY was found to be 97.69, 94.83, 94.83, and 98% under equilibrium conditions of contact time, temperature, adsorbent dose, and TY concentration. Similarly, the uptake capacities of TAC under optimal experimental conditions were found to be 19.56, 18.96, 18.52, and 18.75 mg/g. Thermodynamic studies of TAC, CMC, and UFC showed that the values of ΔG are negative, while those of ΔH and ΔS are positive in all cases and at all temperatures. This indicates that the TY elimination process is endothermic and spontaneous with an entropy-driven nature. The obtained results indicate that the as-fabricated low-cost biomaterials can effectively remove dyes from wastewater through physicochemical interactions. The removal process was influenced by the nature of the adsorbent and the operating variables.

## 1. Introduction

Biopolymers are one of nature’s numerous abundant resources. Cellulose, starch, protein, DNA, etc., are all examples of biopolymers that are generated by living organisms. Cellulose is an example of a semi crystalline polymer. The crystalline parts are the cellulose whiskers, which are used as reinforcing phases in synthetic and natural polymeric matrices due to their high aspect ratio and extensive contact area. The vast specific surface area, high mechanical quality, biocompatibility, and regenerative nature of these materials all contribute to their enduring appeal. The potential uses for these nanofibers in fields such as tissue engineering and nanoelectronics are vast [1]. Cellulose is a valuable construction ingredient found largely in intact wood, but also in natural textile fibers such as cotton and flax, and in paper and board. Artificial cellulose-based threads and films, and various cellulose derivatives used in the food, printing, cosmetic, oil well drilling, textile, pharmaceutical industries, etc., and in daily life, all have their origins in cellulose, which is recognized as a valuable starting material for subsequent chemical transformation [2]. Although the output of the approach may not be practical at larger production scales, plant origin may be preferable due to the availability of vast volumes of waste biomass at low or no cost. Both the crystallinity of the particular plant fibers and the extraction procedure influence the morphological and structural properties of the fibers obtained, such as their entanglement and geometrical dispersion and the extraction yield (the amount of cellulose obtained from a given weight of microfiber). Nano cellulose is often extracted from fibrous vegetable material that is not ideal for use in textiles (such as kenaf core fibers) or is a byproduct of a plant that has no significant use in the textile industry but is useful in other fields (e.g., pineapple leaf fibers). Vegetable material from a plant with no longer important functions or whose usage has finished is another appealing possibility (a large number of herbaceous and leaf fibers fall in these category). Alkali treatment combined with high pressure defibrillation and acid treatment are two methods that have recently been studied for their effects on banana pseudo stem fibers [3]. Glucose repeat units connected by 1, 4-beta-glucosidic linkages make up cellulose, the most abundant polysaccharide found in nature. Cellulose and products made from it are versatile, long-lasting materials. For use in textiles, composites (safety glass), and thermoplastics, cellulose nitrate, cellulose acetate (CA), and cellulose xanthate (rayon) fibers may be readily produced or drawn. Cellulose-based materials allow for more precise control over support design since they are less soluble in water than starch (which has a very similar glucose-based structure but with alpha linkages). Because of the hydrogen bonds formed between the hydroxyl groups, cellulose is highly crystalline, little soluble in water, and resistant to degradation in living organisms. Tissues with more amorphous regions tend to degrade more quickly [4]. By combining functional groups in a controlled manner, we may give our products new, useful properties and expand their potential applications. In such a case, the functionalization process might be executed in a solvent made from unprocessed cellulosic material. The functionalization of cellulose has been utilized in several publications with great success [5]. Within this contribution, we provide two methods for working with amino-group-containing cellulose derivatives. Intermediates of homogeneously generated cellulose were used in amine displacement operations, and the grafting of 2-methyl-1, 3-oxazoline onto cellulose was initiated using these intermediates [6]. Homogeneous cellulose functionalization has long been a focus of cellulose research, despite the fact that heterogeneous methods are used in the synthesis of the majority of commercial cellulose derivatives. Besides the various modification strategies, the thermo-chemical modification of cellulose is also a fruitful approach. This process involves the application of heat and chemicals to cellulose fibers to alter their properties. This modification method has gained significant attention due to its ability to enhance the performance of cellulose-based materials, such as paper, composites, and textiles for various applications. Some of the possible methods for thermo-chemical modification of cellulose include pyrolysis, torrefaction, carbonization, benzylation, and acid hydrolysis, etc. In pyrolysis, cellulose is treated thermally in the absence of oxygen to break down its molecular structure [7]. Similarly, in a mild pyrolysis process known as torrefaction, cellulose is heated at temperatures between 200 and 300 °C in the absence of oxygen. In this process, the volatile components of cellulose can be removed and result in the production of a carbon-rich material with better quality for environmental and energy applications [8]. Carbonization is another high-temperature treatment of cellulose at temperatures above 500 °C in the absence of oxygen to produce a carbon-based material. It was observed that the resulting material acquired desired and controlled properties including high thermal and mechanical stability, better electrical conductivity, and good surface characteristics [9]. Likewise, acid hydrolysis and benzoylation are also among the important chemical treatments that involve the use of acid to break down polymeric cellulose into smaller components and to modify cellulose by introducing some new functionalities [10]. Various industrial effluents discharge toxic dyes to aquatic environments, which affects the life of human beings directly and indirectly. Several damages to humans, such as dysfunction of the liver, reproductive system, kidneys, respiratory and central nervous system, are caused by dyes [11]. It is of utmost importance to remove dyes from wastewater in the textile industry, because they are harmful to the environment even at very low concentrations. The commonly used methods for removing contaminants from wastewater include membrane filtration, flocculation, coagulation, oxidation, and adsorption [12,13]. To address this issue, adsorption is one of the effective techniques used for the treatment of textile wastewater. Aside from the details given above, cellulose and its chemically/thermally modified bio-polymeric form are believed to be useful in the removal of poisonous dyes and heavy metal ions from aqueous systems. In light of previous results [14,15], we believe that the thermal and chemical modification processes influence the structure, surface area, and morphological properties, and thus the adsorption performance of cellulosic materials. Therefore, the aim of this study is to modify raw cellulose using thermal and chemical approaches to remove selected contaminants using adsorption processes. Such treatments may tailor their surface properties to improve their affinity and selectivity for specific adsorbates, thus improving the performance and efficiency of the adsorption process. This study also aims to utilize abundantly available natural biomaterials and simple synthesis methods that can be executed in less sophisticated laboratories. An additional objective is to eliminate the requirement for highly trained scientific personnel and expensive modification/fabrication approaches.

## 2. Results and Discussion

### 2.1. Spectroscopic Analysis of TAC, CMC, and UFC

**Figure 1:**
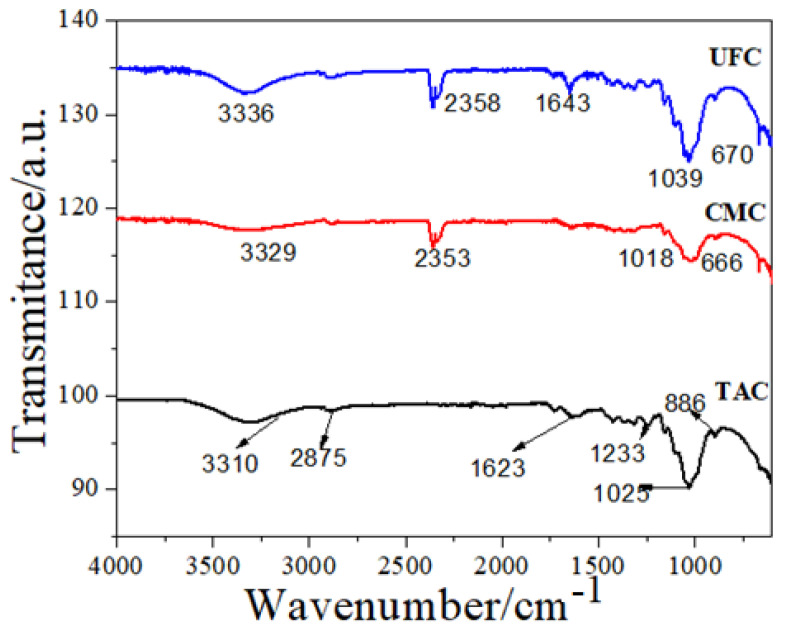
FTIR spectra of unmodified cellulose (UFC), chemically modified cellulose (CMC), and thermally modified cellulose (TAC).

FTIR analysis of unmodified, thermally modified, and chemically modified cellulose (Figure 1) adsorbents was carried out to assess their respective chemical compositions. The spectral peaks at 670, 666, and 886 cm^−1^ were assigned to the C-Cl functional groups for UFC, CMC, and TAC, respectively. The peaks at 1039, 1018, and 1025 cm^−1^ represented the strong stretching of C-OH functional group. The peak at 1233 cm^−1^, usually allocated in thermally functionalized (TAC) cellulose, manifested the strong stretching of the C-O-C functional group. In addition, the peaks at 1643 and 1623 cm^−1^ in UFC and TAC were ascribed to the weak C=C alkene, while the peaks at 2358 and 2353 cm^−1^ in UFC and CMC, respectively, corresponded to C-N functionality. The peak at 2875 cm^−1^ in TAC indicated weak C-H stretching. The peaks at 3336, 3329, and 3310 cm^−1^ were attributed to the strong OH stretching functionality, respectively, in UFC, CMC, and TAC. Overall, the FTIR analysis reflects some qualitative changes upon thermal/chemical modifications of the base material (UF).

### 2.2. Morphology of TAC, CMC, and UFC

**Figure 2:**
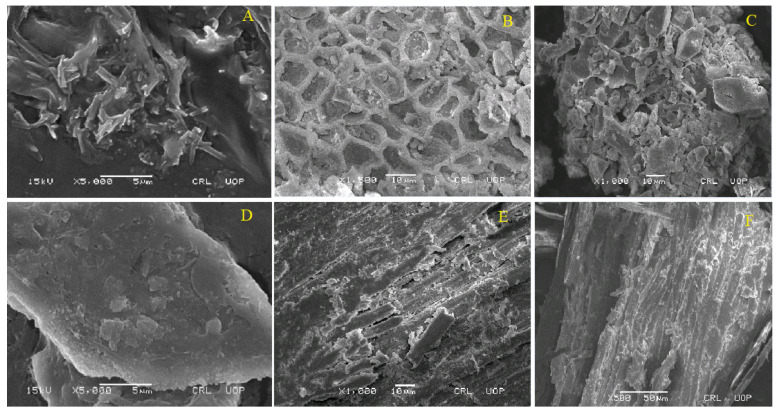
(**A**,**D**) SEM images of unmodified cellulose (UFC) before (**A**) and after adsorption (**D**); (**B**,**E**) SEM images of the chemically modified cellulose (CMC) before (**B**) and after adsorption (**E**); and (**C**,**F**) SEM images of the thermally activated cellulose (TAC) before (**C**) and after adsorption (**F**), respectively.

SEM micrographs of cellulosic materials such as UFC, CMC, and TAC before adsorption of TY are shown in Figure 2A–C. These micrographs show a rather smooth and uninterrupted textured microstructure, especially chemically treated cellulose microphages (Figure 2B), indicating a honeycomb texture. Likewise, the SEM images of UFC, CMC, and TAC after adsorption of TY dye on the surface are shown in Figure 2D–F, respectively. It can be seen that the surfaces of these adsorbents are somewhat rough and cracked after being used to remove TY. Based on FTIR and SEM results, it can be reflected that chemical modification of cellulose via benzoylation reaction has resulted in the addition of some new functional groups onto the cellulose surface through a chemical reaction, which can alter the FTIR peak positions, surface morphology, charge, and hydrophobicity of the original cellulose (UFC) as compared with those of the CMC. Similarly, thermo-modification of cellulose may also lead to the removal of hemicellulose and lignin components, resulting in an increase in crystallinity and surface area of the cellulose UFC. This process can also lead to the formation of new surface functional groups, such as carboxyl and hydroxyl groups. These changes not only altered the surface morphology/porosity and hydrophobicity, but also the FTIR peaks’ position to some extents. These qualitative changes suggest the successful modifications of the raw cellulose (UFC).

### 2.3. Absorption Spectroscopic Analysis of TAC, CMC, and UFC

**Figure 3:**
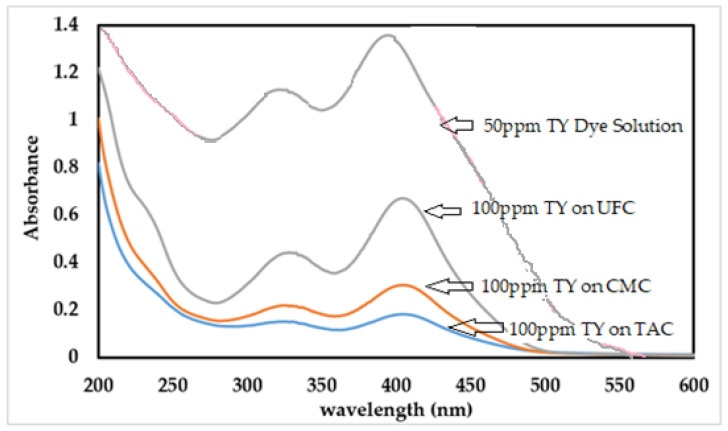
UV-Visible Spectra for a 50 ppm TY dye solution and remaining dye concentration after its adsorption on UFC, CMC, and TAC while using a stock solution of 100 ppm under same experimental conditions.

Figure 3 shows the absorbance of UFC, CMC, and TAC performed for 50 mL of 100 mg/L TY solution for 30 min using 0.1 g adsorbent dose at room temperature. The results shown in Figure 3 clearly demonstrate that TY concentration suddenly drops for the modified cellulose adsorbents, indicating the low adsorption ability of UFC compared with CMC and TAC. The detailed comparison of cellulosic adsorbents was performed for TY dye adsorption performance by varying the operating parameters, including adsorbent dose, initial TY concentration, contact time, and temperature. The difference in the results presented in Figure 3 suggests that thermo- and chemical-modification considerably improved the adsorptive performance of cellulose-based materials. These may be due to an increase in surface area and porosity resulting from thermo-modification, which can provide more active sites for adsorption. Further, the chemical modification introduced some new functional groups that increased the surface charge and hydrophobicity of the material (CMC), which make it more attractive toward TY as compared with the UFC.

### 2.4. Effect of Adsorbent Dose on TAC, CMC, and UFC

**Figure 4:**
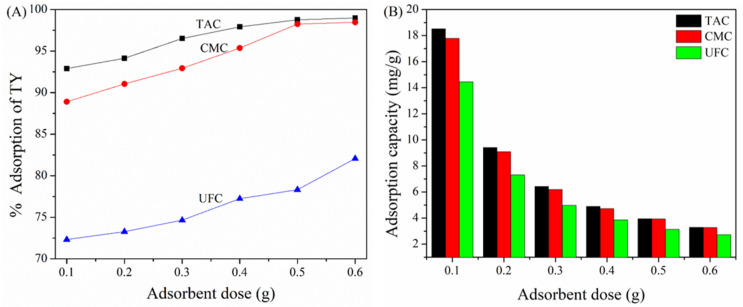
(**A**) Adsorbent dosage affects the % adsorption of TY dye on various adsorbents such as TAC, CMC, and UFC from water solution. (**B**) Adsorption capacity (mg/g) of TY on TAC, CMC, and UFC as a function of adsorbent dosage. Time = 30 min, temperature = 25 °C, and TY starting concentration = 50 mg/L.

The amount of adsorbent used is a key factor regarding the efficiency of dye removal from contaminated water. Figure 4 displays the effect of adsorbent dosage (0.1–0.6 g) on the adsorption of TY on TAC, CMC, and UFC studied using 20 mL of a 50 mg/L TY solution for 30 min at 25 °C. It can be noticed that the percent adsorption of TY onto the surface of various adsorbents increases with increasing the adsorbent dose, irrespective of the adsorbent nature, i.e., 92.5% at 0.1 g to 97.83% at 0.6 g, 87.51% at 0.1 g to 96.89% at 0.6 g, and 72.48% at 0.1 g to 88.08% at 0.6 g for TAC, CMC, and UFC, respectively (Figure 4A). The percentage of dye adsorption is lower at low adsorbent doses but increases for higher doses, indicating an increase in the availability of surface active sites on the adsorbent for a fixed amount of TY in solution. This, in turn, contributes to higher values of % adsorption of dyes on the adsorbent, whereas the adsorption capacity decreases markedly from 18.21 to 3.58 mg/g, 17.51 to 3.01 mg/g, and 14.01 to 2.7 mg/g for TAC, CMC, and UFC, respectively, with an increase in the dosage of respective adsorbent, as shown in Figure 4B. According to the findings, increasing the amount of adsorbent used for the same concentration of dye in a solution results in a decrease in the adsorption capacity, which is measured in mg/g. This suggests that when the surface sites available for adsorption are limited, the adsorption capacity increases as more dye molecules become available for adsorption. However, when the amount of dye is fixed, an increase in adsorbent dose leads to a decrease in adsorption capacity (mg/g), indicating that more adsorption sites are available for a limited amount of dye. Additionally, at the same time, the agglomeration of adsorbent may occur at higher dose which hinders the competition of dye molecules for active sites, eventually resulting in decreasing the adsorption capacity. In other words, the specific adsorption capacity (mg/g) of adsorbents decreases under these conditions. Similar observations for adsorptive removal of heavy metals have been already reported by Tang and co-workers [16].

### 2.5. Effect of Initial TY Concentration on TAC, CMC, and UFC

**Figure 5:**
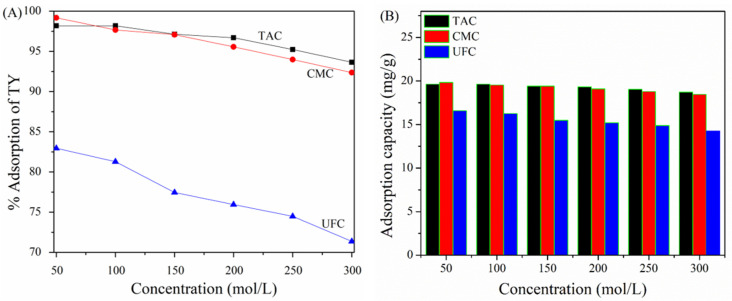
(**A**) The effect of initial TY concentration on % removal of TY from water by using TAC, CMC, and UFC adsorbents. (**B**) The effect of initial TY concentration on TAC, CMC, and UFC adsorption capacity (mg/g) in aqueous medium. Experimental parameters were: time = 30 min, adsorbent dose = 0.1 g, and temperature = 25 °C.

Figure 5 demonstrates the influence of the initial TY concentration on adsorption/removal performances of TAC, CMC, and UFC, examined using 0.1 g of adsorbent dose in 20 mL of different initial concentrations of TY solution (i.e., 50, 100, 150, 200, 250, and 300 mg/L) at room temperature (25 °C). The results presented in Figure 5A,B reveal that both the removal efficiencies and adsorption capacities of TAC, CMC, and UFC decrease as the initial TY concentration increases from 50 to 300 mg/L. This corroborates with the results reported in the literature [17,18], indicating that increasing the adsorbate concentration gradually increases the resistance to the mass-transfer between liquid/solid phases, resulting in a decline in the adsorption performance. The variation in the observed trends is prominent in the case of UFC compared with other adsorbents, which highlights that surface modification has significantly activated the cellulosic adsorbent [19].

### 2.6. Effect of Contact Time on Adsorption of TY on TAC, CMC, and UFC

**Figure 6:**
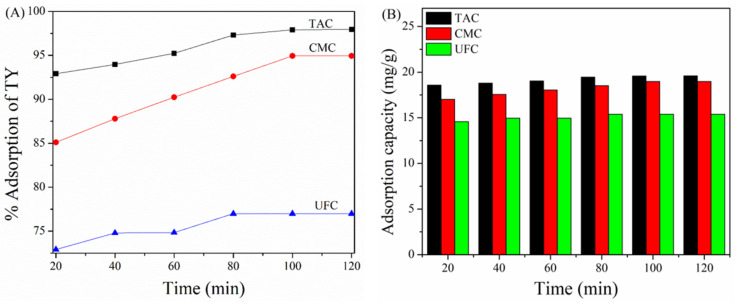
(**A**) The effect of contact time on the (%) removal efficiency of TY dye from aqueous medium using various adsorbents TAC, CMC, and UFC. (**B**) Effect of contact time on adsorption capacity of TAC, CMC, and UFC. Experimental parameters: temperature = 25 °C, adsorbent dose = 0.1 g, initial TY concentration = 50 mg/L.

Adsorption experiments were performed using 20 mL of a 100 mg/L TY solution and 0.1 g adsorbent doe at a stirring speed of 240 rpm and a temperature of 25 °C for a contact time in the range of 20–120 min with a 20 min interval, as shown in Figure 6. The results (Figure 6A) indicate that TAC and CMC exhibit remarkably higher removal rates of TY, reaching 97.96% and 94.94% compared with that of the UFC (76.99%). The tendency of TY uptake gradually increases with increasing the contact time until 80 min for UFC, 100 min for CMC, and 120 min for TAC followed by nearly constant values, indicating that adsorption equilibria has been reached at/or beyond that time. Figure 6B demonstrates that the relative adsorption capacities of TAC, CMC, and UFC are 19.59, 18.98, and 15.39 mg/g, respectively. The higher adsorption capacities of TAC and CMC are due to the higher proportion of active sites that are formed due to their surface modification. Similar observations of enhanced adsorption capacity due to the surface modification of adsorbents has been reported in the literature [20].

### 2.7. Effect of Temperature on Adsorption of TY on TAC, CMC, and UFC

**Figure 7:**
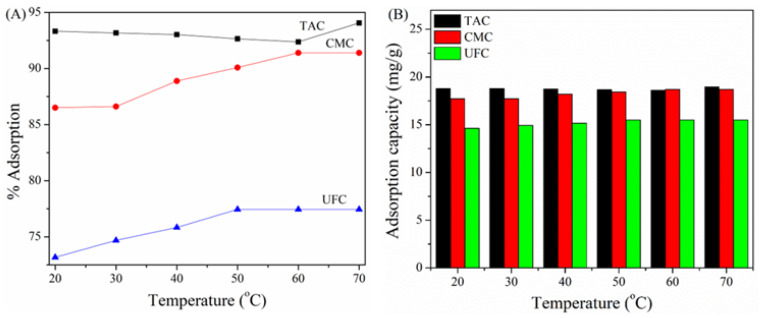
(**A**) Temperature effect on TY dye removal efficiency (%) from TAC, CMC, and UFC in aqueous solution. (**B**) The effect of temperature on the adsorption capacity (mg/g) of TY dye on TAC, CMC, and UFC from aqueous solution. The experimental conditions were as follows: contact time = 30 min, adsorbent dose = 0.1 g, initial TY concentration = 50 mg/L.

The removal of TY has been also investigated as a function of medium temperature (20, 30, 40, 50, 60, and 70 °C) for different adsorbents TAC, CMC, and UFC using a fixed dose of each adsorbent (0.1 g) for 50 mg/L dye concentration at 30 min contact time. Figure 7A shows that the removal efficiencies of TY using the three adsorbents increases with increasing temperature from 95.5% at 20 °C to 96.83% at 70 °C, 84.15% at 20 °C to 93.49% at 70 °C, and 71.5% at 20 °C to 77.15% at 70 °C, for TAC, CMC, and UFC, respectively. The adsorption capacities of TAC, CMC, and UFC, as illustrated in Figure 7B, were also found to increase with an increase in temperature, which reflects the endothermic nature of the adsorption process. In this case, the rise in temperature not only enhances the active adsorption sites on the surface of adsorbents, but also favors both the mobility of ionic species and mass transfer process from the bulk of solution to the solid surface of materials/adsorbent [21].

### 2.8. Adsorption Kinetic Studies

**Figure 8:**
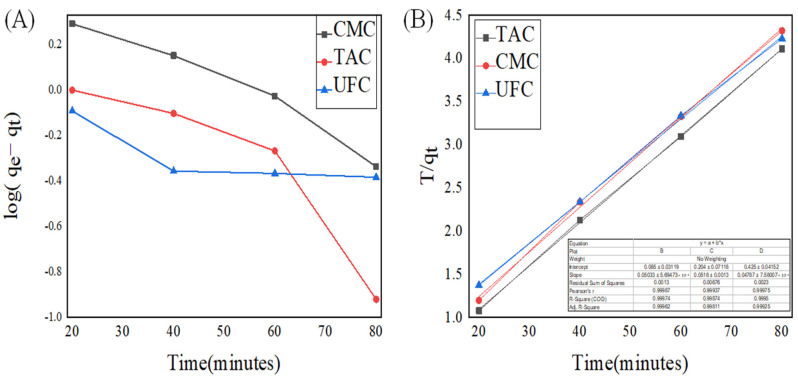
(**A**) Pseudo-first order kinetics model for the adsorption of TY on various adsorbents TAC, CMC, and UFC. (**B**) Pseudo-second order kinetics model for TY adsorption on various adsorbents such as TAC, CMC, and UFC.

The adsorption kinetics provides insightful information about the mass transfer of adsorbate from liquid medium to the solid adsorbent surface. The kinetic studies of TY adsorption on TAC, CMC, and UFC were carried out using 50 mg/L TY solution (50 mL) at room temperature and 0.1 g of adsorbents. The obtained data were then fitted to pseudo-first order (PFO) and pseudo-second order (PSO) models (Equations (1) and (2)) [22]:

(1) $$log\left(q_{e}-q_{t}\right)=logq_{e}-\frac{k_{1}t}{2.303}\;\text{(PFO)}$$

(2) $$\frac{t}{q_{t}}=\frac{1}{2kq_{e}^{2}}+\frac{t}{q_{e}}\;\text{(PSO)}$$

where *q_t_*, and *q_e_* are the adsorption capacity (mg/g) at time’s interval at equilibrium, while *k*_1_ and *k*_2_ are the pseudo-first order rate constant (min^−1^) and pseudo-second order rate constant (g/mg/min), respectively. The values of rate constants (*k*_1_ and *k*_2_) were obtained from the linear plots of log (*q_e_ − q_t_*) versus time for PFO and *t/q_e_* versus time for PSO, respectively, as presented in Figure 8A,B. It is clear that PFO is relatively poorly fitting to the experimental data with an R^2^ value of 0.964 compared with the PSO model, which presents best fitting of the experimental data with high R^2^ (0.996) and close matching the theoretical *q_e_* values with experimental *q_e_*, as shown in Table 1. The obtained results indicate that physicochemical adsorption of TY takes place on TAC, CMC, and UFC following pseudo-second order kinetics to an appreciable extent.

### 2.9. Adsorption Isotherm Model

The experimental adsorption data of TY onto different adsorbents TAC, CMC, and UFC were investigated by means of Langmuir, Freundlich, and Temkin isotherms (Equations (3)–(5)).

**Figure 9:**
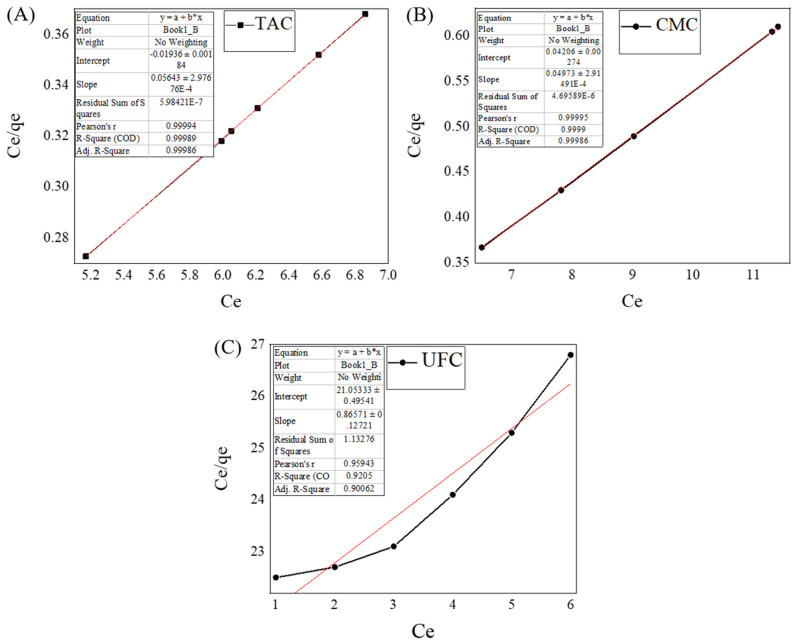
Langmuir isotherm model for adsorption of TY dye on (**A**) TAC, (**B**) CMC, and (**C**) UFC adsorbents.

Langmuir adsorption isotherm presumes that adsorption takes place as a monolayer over identical active sites (homogenous surface), which is mathematically expressed as follows [23]:

(3) $$\frac{C_{e}}{q_{e}}=\frac{C_{e}}{q_{m}}+\frac{1}{q_{m}K_{L}}$$

where *C_e_, q_e_*, *q_m_*, and *K_L_* are the equilibrium concentration of TY, equilibrium adsorption capacity, maximum adsorption capacity, and Langmuir constant, respectively. The linearized plot of *Ce/q_e_* versus *Ce* (Figure 9A–C) provides *q_m_* and *K_L_*, which refer to the potency of adsorbate–adsorbent interactions.

**Figure 10:**
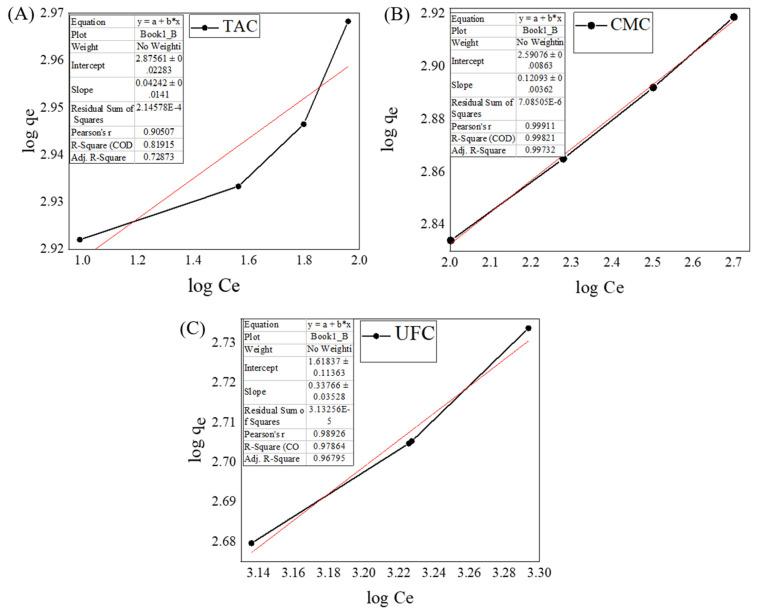
Freundlich isotherm model for adsorption of TY dye on (**A**) TAC, (**B**) CMC, and (**C**) UFC adsorbents.

The Freundlich adsorption isotherm model describes the physical nature of adsorption as a multilayer phenomenon, which is mathematically expressed as:

(4) $$logq_{e}=logK_{F}+\frac{1}{n}logC_{e}$$

where *q_e_* is the adsorption capacity (mg/g), *K_F_* is Freundlich adsorption capacity mg/g, and *n* is the intensity constant. The representative plots of the Freundlich isotherm model for the adsorption of TY dye on TAC, CMC, and UFC adsorbents are given in Figure 10A–C.

**Figure 11:**
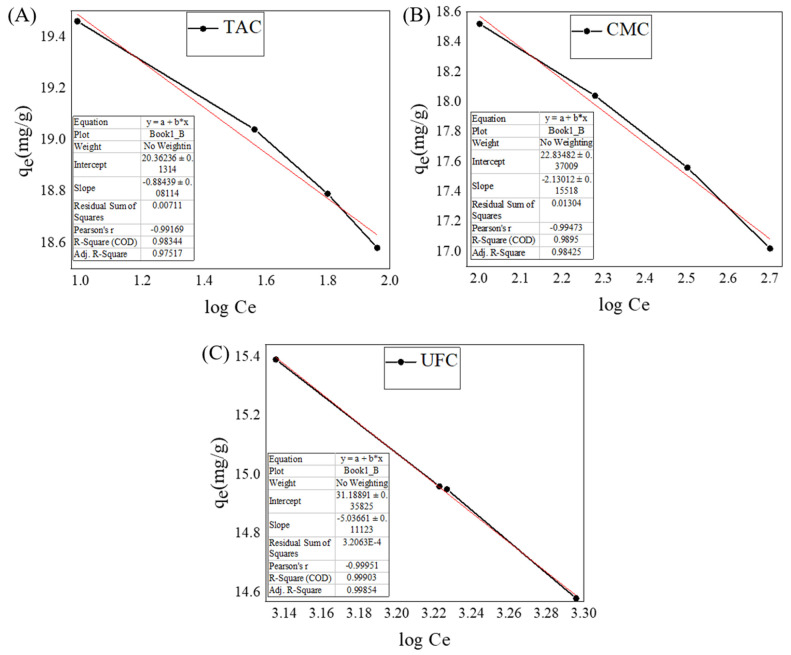
Temkin isotherm model for adsorption of TY dye on different adsorbents (**A**) TAC, (**B**) CMC, and (**C**) UFC.

The Temkin adsorption model highlights the adsorption on relatively heterogeneous surfaces of adsorbent. It also gives information on adsorbate/adsorbent interactions and enthalpy of adsorption, which decreases with the progress of the adsorption process, as shown by following equation:

(5) $$q_{e}=Blnk_{TM}+BlnC_{e}$$

where *K_Tm_* and *B* are Temkin’s adsorption constants, with *K_Tm_* relating to maximal binding energy (mg/L) and *B* relating to heat of adsorption. The slope and intercept of the linear plots of *q_e_* versus *log C_e_* are used to determine these values. The Temkin plots for the adsorption of TY dye on different adsorbents TAC, CMC, and UFC are shown in Figure 11A–C.

The results of the above-mentioned isotherm models are presented in Table 2. The results clearly show that all these models fit the adsorption equilibrium data, but the best fit isotherms are Langmuir (Figure 9) and Temkin (Figure 11) as compared with the Freundlich (Figure 10). This trend can be further seen from the corresponding higher R^2^ values, i.e., 0.990 and 0.983 for Langmuir and Temkin, respectively, and the relatively lower value of R^2^ (0.818) for the Freundlich isotherm. Adsorption on the surface of TAC, CMC, and UFC manifests relatively heterogeneously, as shown by the Temkin isotherm, and most of the TY dye is removed dominantly through chemisorption mechanism, as indicated from the outcomes of the Langmuir isotherm. Based on kinetics and isothermal results, overall, it is demonstrated that the adsorption process is not purely chemical or physical, but that it can be of a physicochemical nature. These possible physicochemical interactions may include Vander Waals forces, hydrogen bonding, and electrostatic interactions between TY molecules and the surface of these adsorbents.

**Figure 12:**
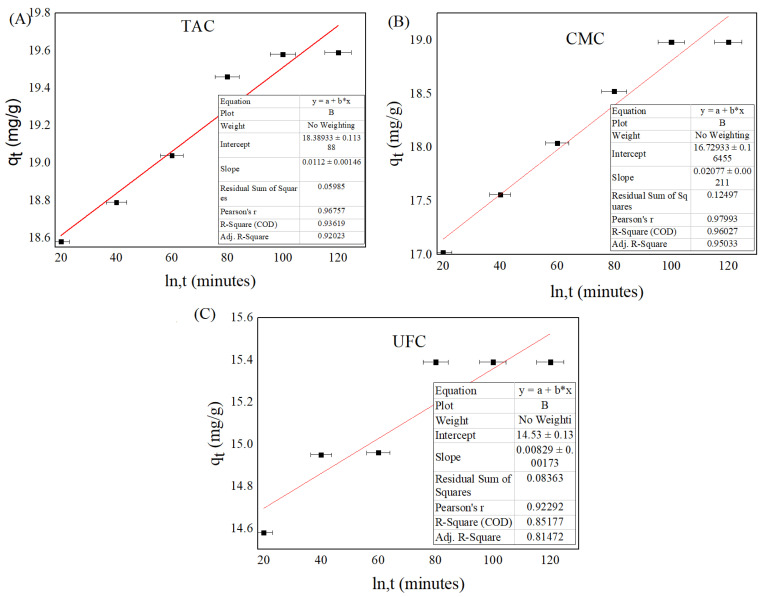
Elovich Kinetic model for adsorption of TY dye on different adsorbents (**A**) TAC, (**B**) CMC, and (**C**) UFC.

The simplified form of the Elovich equation (Equation (6)) was utilized to determine the chemisorptive nature of TY adsorption on the surface of cellulosic adsorbents such as TAC, CMC, and UFC [24].

(6) $$\;q_{t}=\frac{1}{\beta }\ln\left(\alpha \beta t+1\right)\;\text{nonlinear}$$

(7) $$q_{t}=\frac{1}{\beta }\ln\left(\alpha \beta \right)+\frac{1}{\beta }lnt\;\text{linear }$$

where α is the adsorption rate (mg/g min), β is the desorption constant (g/mg), while q_t_ is the amount adsorbed at time t (mg/g). Here, we have optimized both linear and nonlinear equations of the Elovich adsorption model. For the nonlinear model, a user define equation (*q_t_* = 1/*B* × (ln(*A* × *B* × *t* + 1)) was applied in which *A* and *B* represent *α* and *β*, respectively. For the linear equation, we fitted the data directly in origin through the help of Equation (7), and the results are presented in Figure 12. It can be seen that the TY removal process through all the three adsorbents obeys the Elovich model with reasonable R^2^ values in the range of 0.85 and 0.96. The results are consistent with the reported literature [25]. Based on these results, it is suggested that the adsorption might be chemisorption corresponding to the heterogeneous nature of the active sites.

### 2.10. Recycling and Reusability of the Materials

**Figure 13:**
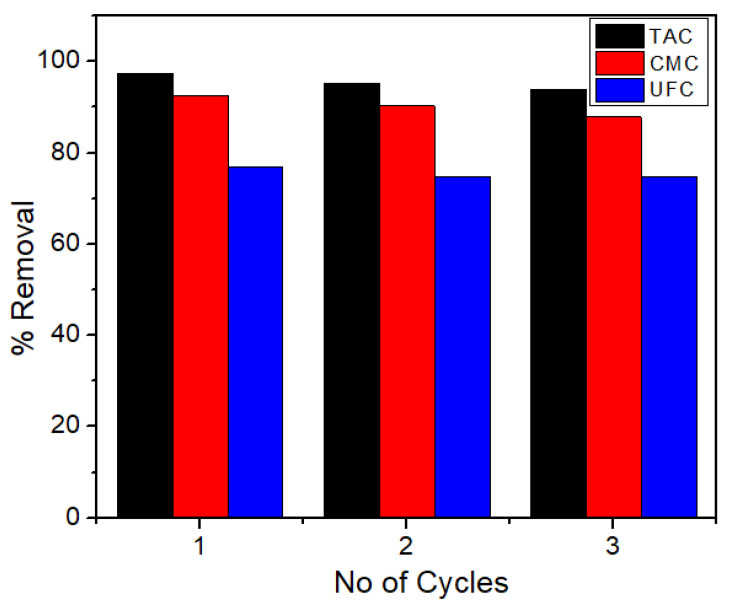
Recycling performance of UFC, CMC, and TAC toward the removal of TY dye from water solution while using similar experimental conditions such as time = 30 min, temperature = 25 °C, and initial concentration of TY = 50 mg/L.

The stability, reusability, and recycling performance of UFC, CMC, and TAC toward the removal of TY dye from water solution were also tested using similar experimental conditions, such as time = 30 min, temperature = 25 °C, and initial concentration of TY = 50 mg/L, and the results are presented in Figure 13. In a general recycling experiment, the desired adsorbent material was recovered after each cycle, washed with water, and dried in an oven at 100 °C for an hour before being reused in the TY dye removal process. The percentage of dye removed was then calculated and plotted as a function of the number of cycles. It was observed that the recycling and reusability efficiency of the material decreased slightly over three consecutive cycles. This minor decrease in efficiency may have been due to either material loss or a slight modification to the surface of the materials. However, the overall recycling efficiency remained satisfactory, indicating the favorable performance of the materials used in the current experimental conditions.

### 2.11. Adsorption Thermodynamics Studies

A thermodynamics study of TY adsorption on TAC, CMC, and UFC was also investigated to calculate the change in entropy (ΔS), change in enthalpy (ΔH), and Gibbs free energy (ΔG) by using the following equations (Equations (8)–(10)) [26]. First of all, we calculated the thermodynamics distribution coefficient (*K_d_*) or equilibrium distribution constant (*K_eq_*) by using Equation (8a) at different temperatures.

(8a) $$K_{d}=\frac{C_{adsorbed}}{C_{e}}$$

where *K_d_* or *K_eq_* is the measure of the affinity of an adsorbent/sorbent for a particular adsorbate, which is TY dye in this case. Quantitatively, it is simply the ratio of concentration of the TY (adsorbate) adsorbed onto the sorbent (*C_adsorbed_*) to the concentration of the adsorbate remaining un-adsorbed in the bulk of the solution (*C_e_*) at equilibrium. Similarly, using the values of *K_eq_*, the values of ΔG were estimated through Equation (8b) [27].

ΔG = −RT ln*K_eq_* (8b)

(9) $$\ln K_{eq}=\frac{\Delta H}{\mathrm{RT}}-\frac{\Delta S}{R}$$

In this Equation (9) ΔH, R, ΔS, T, and K are the enthalpy change, universal gas constant, entropy change, temperature in Kelvin, and adsorption constant, respectively. The linearized plot of ln*K* versus 1/T provides the values of ΔS and ΔH. Similarly, these values were also cross-checked using Equation (10).

ΔG = ΔH − TΔS
(10)

**Figure 14:**
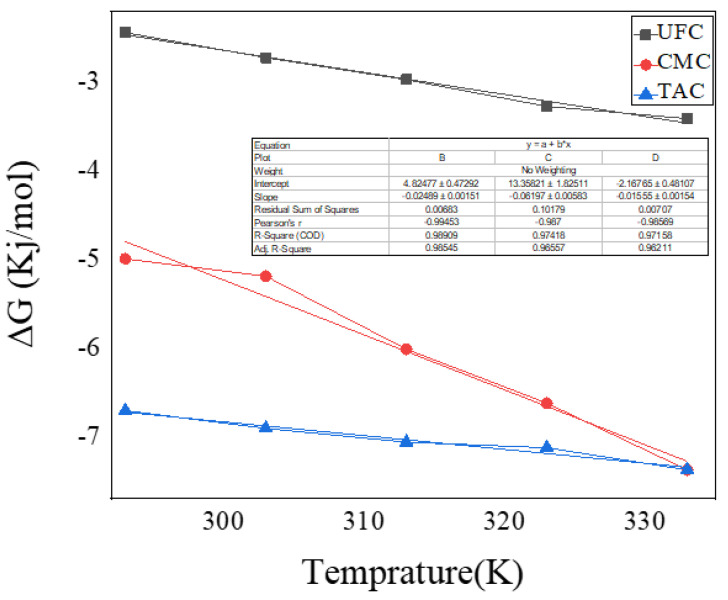
ΔG vs. Temperature (T) plots for adsorption of TY dye on different adsorbents such as on TAC, CMC, and UFC adsorbents.

Figure 14 and Table 3 report the values of ΔH, ΔS, and ΔG. It is observed that the ΔG values are negative at all temperatures, even below room temperature. The negative ΔG value predicts the spontaneity of the adsorption process. Furthermore, ΔH and ΔS values are positive, reflecting that the adsorption of TY on TMC, CMC, and UMC is spontaneous, endothermic, and entropy-driven in nature. Additionally, the positive ΔS value indicates that the movement of molecules at the water/solid interface is more as compared with their movement in the bulk of solution. The positive ΔH further reflects that the transfer of dye molecules from the bulk of the solution to the solid surface requires some energy to overcome the dye–water interactions. It is because sulfonate groups on TY make it a highly polar molecule; as a result, the dye molecules can have strong electrostatic interaction with water. This is also reflected by an increase in the (−ΔG) values with an increase in temperature. Thus, temperature facilitated the adsorption in the present case. In other words, the TY dye adsorbed onto TMC, CMC, and UMC followed physisorption at the surface of adsorbent. This means that the removal of TY from water with the help of these materials is an entropy-driven spontaneous process. Based on thermodynamics and kinetics results, it is supposed that TY can interact with the cellulose-based materials through common physicochemical interactions such as Vander Waals forces, hydrogen bonding, and electrostatic interactions. In addition, due to the presence of carboxyl and hydroxyl groups at the surface of cellulose-based adsorbents, it is expected that these materials may be protonated and become positively charged at low pH, while at high pH (alkaline pH), the surface becomes negatively charged due to possible deprotonation. Moreover, comparative evaluations of the dye (TY) removal abilities of three cellulose-based materials, namely, unmodified cellulose (UFC), chemically modified cellulose (CMC), and thermally treated/activated cellulose (TAC), indicate that modifications through thermo- and chemical-processing have significantly enhanced the adsorption performance of the cellulose-based materials. The improvements may be attributed to an increase in surface area and porosity resulting from thermo-processing, which provides more active sites for adsorption. Additionally, chemical modification has introduced new functional groups that have increased the surface charge and hydrophobicity of the material (CMC), thereby rendering it more appealing for TY compared with UFC.

A concise comparison of the TY dye removal performances of the present materials (UFC, CMC, and TAC) with the data of some of the selected materials, reported previously, is also presented in Table 4. It is evident that all three types of materials, i.e., the un-functionalized (UFC), thermally modified (TAC), and thermo-chemically modified (CMC) materials, have appreciable and better dye removal efficiency compared with the other materials reported in the literature. It is noted from the overall results that the adsorption efficiency of the different experiments varies with the changes in the experimental conditions such as type and amount of material/adsorbent, pollutant concentration, contact time, as well as pH and temperature of the medium. In addition, the chemical composition, different textures, and morphological characteristics of the adsorbent significantly affect its efficiency. It is observed that the present materials, which are obtained via inexpensive and easily accessible approaches from naturally available cellulose, possess attractive outcomes. Hence, it is also important to fabricate materials with a good balance of structural, morphological, and chemical properties for decontamination of wastewater with an inexpensive and simple approach.

## 3. Experimental Methods

### 3.1. Materials Required

**Scheme 1:**
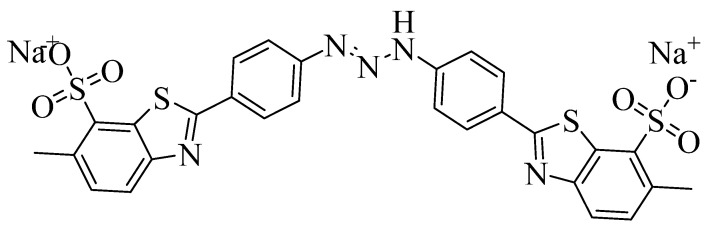
Chemical structures of Titan Yellow dye.

The textile dye Titan Yellow (TY) and all required chemicals including Potassium Hydroxide, Sodium Hydroxide, Ethylene Diamine, Benzoyl Chloride, and Thionyl Chloride were purchased from Sigma Aldrich (Shanghai, China) in analytical grade and were used without further purification. The chemical structures of Titan Yellow dye is given in Scheme 1. Distilled water was used for solutions’ preparation in all the experiments. The shot stem of *Olive Europa* was collected from the valleys of district Malakand, Khyber Pakhtunkhwa, Pakistan, cleaned and washed with distilled water, dried in oven at a temperature of 50 °C for 2 h, and used for cellulose extraction as per the following procedure.

### 3.2. Preparation and Functionalization of the Adsorbents

The 3 cm long dried shot stems of *Olive Europa* were soaked in 3 L of tap water for 45 days at room temperature; as a result of this, the fibers were clearly separated out from sticks. The extracted fiber was washed with distilled water followed by drying in an oven at 110 °C for 3 h. The dried fibers were subjected to Soxhlet extraction [33] with n-hexane for 72 h and then washed several times with distilled water. The obtained material was divided into three proportions for different modes of surface modification such as thermal, chemical, and unmodified, and therefore the resultant fibrous material was labelled accordingly as thermally modified cellulose (TAC), chemically modified cellulose (CMC), and unmodified cellulose (UFC) [34]. 

### 3.3. Thermo-Chemical Modification of Fibers

The chemical activation of cellulose fibers was performed via benzoylation [35] to obtain CMC. The fibers underwent a 48 h drying period at room temperature after being processed for 240 min with a 10% 0.25 M NaOH solution, neutralized with 2% acetic acid, and rinsed once more with distilled water. The dried fibers were then treated with various concentrations of benzoyl chloride solution after being re-soaked in 10% solution of 0.25 M NaOH. The resulting fibers were then washed with distilled water, dried in the air, and then immersed in 10% ethanol for 1 h. In order to obtain the thermally modified cellulose (TAC), the UFC material was kept in furnace at 400 °C in the absence of oxygen for two hours, which resulted in blackish type products named as TAC. The three types of cellulose-based materials (i.e., TAC, CMC, and UFC) were then utilized as absorbents for the removal of Titan Yellow (TY) dye from an aqueous solution while changing various experimental variables [36].

### 3.4. Adsorption Studies

A 1000 mg/L stock solution of aqueous TY was diluted to prepare the desired working concentrations of 50, 100, 150, 200, 250, and 300 mg/L. The comparative adsorption performance of unmodified, thermally modified, and chemically modified cellulosic adsorbents were investigated for TY uptake from aqueous solution by batch experiments. The influence of experimental variables was investigated using 20 mL of TY dye solution with different initial concentrations (50–300 mg/L), adsorbent amounts (0.1–0.6 g), and at room temperature. The kinetics of TY adsorption for different initial concentrations (50–300 mg/L) was performed using 0.1 g adsorbent (fixed amount) in 20 mL dye solution at neutral pH and room temperature for a contact time ranging from 20 to 120 min. The adsorption isotherm and thermodynamics studies were carried out using a fixed amount of adsorbent (0.1 g) in 20 mL of 50 mg/L TY solution at neutral pH for 30 min contact time at different working temperatures (i.e., 20, 30, 40, 50, 60, and 70 °C ). Aliquots of dye solution were withdrawn at regular intervals for each experiment, filtered out, followed by measuring the absorbance of solution at 403 nm (λ_max_) wavelength using a UV-Visible spectrophotometer (Perkin Elmer; Lambda-25, Waltham, MA, USA). A similar set of experiments was carried out individually for three different kinds of adsorbents (i.e., TAC, CMC, and UFC). Ultimately, the obtained results were compared for optimizing the best experimental conditions and adsorbent for TY uptake.

The adsorbed amount *q_e_* (mg/g) of TY and its removal capacity (%) were calculated using the following, Equations (11) and (12):

(11) $$q_{e}=\frac{(C_{0}-C_{e})\times V}{w}$$

(12) $$\%\mathrm{Removal}=\frac{(C_{0}-C_{e})\;}{C_{0}}\times 100$$

where *C*_0_ and *Ce* (mg/L) are initial and equilibrium concentrations of TY, respectively, V is the solution volume (L), and w is the adsorbent amount used (g).

## 4. Conclusions

In summary, we have successfully applied well-known and abundantly available materials by employing inexpensive and easily accessible approaches to modify natural biopolymer to un-functionalized cellulose (UFC), thermally activated cellulose (TAC), and thermo-chemically modified cellulose (CMC). The as-obtained materials were employed for the removal of Titan Yellow dye from aqueous media through a batch adsorption process under various experimental conditions for each adsorbent such as contact time, temperature, concentration, and dosage used. Based on FTIR, SEM, and respective adsorptive results/performance of UFC, CMC, and TAC, it can be reflected that the chemical modification of cellulose via benzoylation reaction, resulted in the addition of some new functional groups onto the cellulose surface through a chemical reaction, which altered the FTIR peak positions, surface morphology, charge, and hydrophobicity of the original cellulose (UFC) as compared with those of the CMC. Similarly, thermo-modification of cellulose may also lead to the removal of hemicellulose and lignin components, resulting in an increase in crystallinity and surface area of the cellulose UFC. This process can also lead to the formation of some new possible surface functional groups, such as carboxyl and hydroxyl groups. These changes have not only altered the surface morphology/porosity and hydrophobicity, but also the dye removal performances. The adsorption rate and capacity of these materials were found to be appreciable, with TAC exhibiting the highest percentage of dye adsorption under present experimental conditions. The materials also showed promising potential for reusability, as observed through their slightly decreased efficiency over three consecutive cycles. Based on thermodynamics and kinetics results, it is supposed that TY can interact with cellulose-based materials through common physicochemical interactions such as Vander Waals forces, hydrogen bonding, and electrostatic interactions. The results obtained in this study suggest that the investigated materials exhibit promising potential for further exploration in the field of water treatment. It is suggested that these materials could be utilized for the removal of contaminants and pathogens from wastewater, providing an environmentally sustainable and economically feasible approach to water treatment.

## Figures and Tables

**Table 1:** The parameter values of pseudo-first order model and pseudo-second order kinetics model for adsorption of TY on TAC, CMC, and UFC.

Adsorbent	Pseudo-First Order			Pseudo-Second Order			Experimental *q_e_*
	*k*_1_ (min^−1^)	R^2^	*q_e_* (mg/g)	*k*_2_ (g/mg/min)	R^2^	*q_e_* (mg/g)	(mg/g)
TAC	0.0336	0.834	2.566	0.0297	0.999	19.88	19.59
CMC	0.0237	0.964	3.439	0.0157	0.998	19.15	18.98
UFC	0.0155	0.033	1.008	0.0024	0.906	18.00	15.39

**Table 2:** Parameters of^.^ Langmuir, Freundlich, Temkim, and Elovich adsorption Isotherm Models for Adsorption of TY Dye on TAC, CMC, and UFC from Aqueous Media.

Adsorption Isotherm Models	Parameters	TAC	CMC	UFC
Langmuir	q_m_ (mg/g)	19.56	20.16	19.99
	K_L_ (L mg^−1^)	3.663	1.138	0.012
	R^2^	0.990	0.999	0.999
Freundlich	n	23.58	8.298	0.0003
	K_F_ (L mg^−1^)	751.1	390.9	44.30
	R^2^	0.818	0.998	0.985
Temkin	b (j/mol)	−0.384	−0.924	−2.187
	K_KT_ (L/mg)	−23.00	−10.72	−6.191
	R^2^	0.983	0.989	0.999
Elovich	α (mg/g min)	18.389	16.729	14.530
	β (g/mg)	0.0112	0.0207	0.0082
	R^2^	0.936	0.960	0.851

**Table 3:** Thermodynamics parameter for adsorption of TY dye on different adsorbents TAC, CMC, and UFC.

Temperature (°C)	ΔG (kJ/mol)			Distribution Coefficient (K_eq_ or K_d_)		
	TAC	CMC	UFC	TAC	CMC	UFC
20	−6.706	−4.993	−2.443	15.674	7.771	2.728
30	−6.907	−5.189	−2.725	15.528	7.849	2.951
40	−7.062	−6.013	−2.974	15.103	10.086	3.139
50	−7.124	−6.622	−3.274	14.197	11.787	3.434
60	−7.220	−7.375	−3.413	13.577	14.360	3.434
	ΔH kJ/mol			ΔS kJ/mol.K		
	TAC	CMC	UFC	TAC	CMC	UFC
	3.107	13.35	4.824	0.012	0.062	0.025

**Table 4:** Comparison of adsorption performance of other adsorbents for TY.

Adsorbent	Dye	Experimental Conditions	Adsorption Capacity (mg/g)	Reference
Modified cellulose lignin	TY	Adsorbent dose: 0.1 g, t: 80 min, Conc: 100 ppm, Sol: 50 mL, T: 25 °C, pH: 9	17.72	[28]
Rice husk modified (KMRHC)	TY	Adsorbent dose 0.1 g, t: 90 min, Conc: 100 ppm, Sol: 50 mL, T: 40 °C, pH: 9	28.02	[29]
Aloe vera leaves wastes-based sulfuric acid modified activated carbon (AV-SAC)	TY	Adsorbent dose: 0.1 g, t: 100 min, Conc: 100 ppm, Sol: 50 mL, T: 40 °C, pH: 8	11.92	[30]
Kahwa tea (KTC)	TY	Adsorbent dose: 0.1 g, t: 180 min, Conc: 100 ppm, Sol: 50 mL, T: 40 °C, pH: 5	7.04	[31]
Chitosan hydroxyapatite nanocomposites (CNNc)	TY	Adsorbent dose: 0.01 g, t: 60 min, Conc: 100 ppm, Sol: 50 mL, T: 25 °C, pH: 7	14.5	[32]
Thermal Activated Cellulose (TAC)	TY	Adsorbent dose: 0.1 g, t: 80 min, Conc: 100 ppm, Sol: 50 mL, T: 25 °C, pH: 7	19.46	This study
Un-Functionalized Cellulose (UFC)	TY	Adsorbent dose: 0.1 g, t: 80 min, Conc: 100 ppm, Sol: 50 mL, T: 25 °C, pH: 7	15.39	This study
Chemically Modified Cellulose (CMC)	TY	Adsorbent dose: 0.1 g, t: 80 min, Conc: 100 ppm, Sol: 50 mL, T: 25 °C, pH: 7	18.52	This study

## Data Availability

Not applicable.

## Sample Availability

Samples of the compounds are available from the authors.
